# Assessment of proton versus photon therapy in the reduction of lymphopaenia in thoracic cancers: A scoping review

**DOI:** 10.1016/j.tipsro.2026.100396

**Published:** 2026-04-01

**Authors:** Erin Meagher, Michelle Leech

**Affiliations:** a Applied Radiation Therapy Trinity, Discipline of Radiation Therapy, Trinity College Dublin, Ireland; b Trinity St. James’s Cancer Institute, Dublin, Ireland

**Keywords:** Lymhopaenia, Proton beam therapy, Thoracic malignancies

## Abstract

**Background:**

Lymphopaenia is characterised by a substantial decrease in lymphocytes, and is experienced by patients receiving radiation therapy for lung and oesophageal cancers. Tumour control and overall survival may be negatively impacted by lymphopaenia. Because of dosimetric benefits which reduce healthy tissue exposure, proton therapy may potentially reduce the prevalence of lymphopaenia, compared to photon therapy.

The purpose of this scoping review was to evaluate the difference in lymphopenia experienced by patients treated with photon or proton therapy for lung and oesophageal cancers. It assessed the incidence and severity of radiation-induced lymphopenia for each treatment modality and dose to lymphoid organs.

**Methodology:**

A systematic search of PubMed, EMBASE, and Web of Science was conducted. The review included both patients who developed lymphopaenia and those who did not, allowing for comparative analysis.

**Results:**

Proton therapy was associated with reduces rates of severe lymphopaenia (Grade ≥ 3), compared with photon therapy. Evidence linking reduced lymphopaenia and overall survival varied, with some studies indicating benefits, while others found no difference.

**Conclusion:**

Proton therapy demonstrated an advantage in minimising lymphopaenia compared to photon therapy. There is a need to establish dose volume constraints for lymphoid organs and determine the impact of lymphopaenia on survival.

## Highlights

• Lymphopaenia is reported as impacting on overall survival in thoracic malignacies.
• Reduction of radiation-induced lymphopaenia is warranted in the immunotherapy era.
• Proton beam therapy may assist in the reduction of lymphopaenia in thoracic malignancies.

## Introduction

Radiation therapy (RT) is a primary treatment modality for thoracic cancers, and while it is highly effective, it can have a detrimental effect on both immunosuppressive and immune activation pathways [1]. In the thoracic region, lymphopaenia- a decrease in lymphocytes, the immune cells critical for anti-tumour activity, is a significant immunological impact of radiation therapy. The depletion of lymphocytes within or passing through the radiation field can occur directly from radiation exposure or through the circulatory system [2]. Lymphopaenia is a common side effect of thoracic radiation which can affect patient outcome including overall survival, particularly for those with severe Grade 4 [3]. Lymphocytes, including T cells, B cells, and NK cells, are essential for maintaining immune surveillance and balance [4].

There is an increasing concern regarding radiation-induced lymphopaenia and its effects on the prognosis of patients receiving RT to the thorax. Identifying modalities which can minimise radiation-induced lymphopaenia (RIL) is of interest as it could affect overall survival. Proton therapy is a promising alternative to photon therapy for this specific purpose [5]. The dosimetric properties of proton and photon therapy vary greatly, which can affect the dose to lymphocytes in target area. Proton therapy delivers radiation to the target with a rapid dose fall-off, theoretically resulting in superior sparing of healthy tissue than photons [6]. This reduces exposure to surrounding lymphoid tissue, thereby potentially reducing the risk of lymphopaenia [5].

**Fig. 1:**
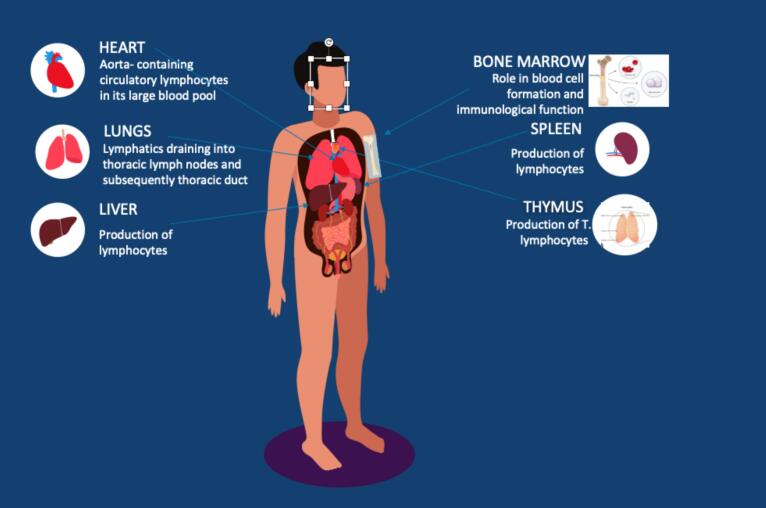
Lymphoid organs.

Key lymphoid tissues and organs in the thoracic region are susceptible to radiation exposure. The spleen, mediastinum- containing lymph nodes, thymus (crucial for T-cell development) and bone marrow, particularly red bone marrow given its role in blood cell formation, are significant normal tissues associated with immunological function [7]. By measuring radiation exposure to these lymphatic and hematopoietic systems, i.e. the estimated dose to immune cells, it is possible to better understand and reduce the effects of radiation-induced lymphopaenia, assisting with treatment modality selection. The effective dose to immune cells (EDIC) approach offers a mechanism to measure the radiation dose to lymphocytes. EDIC focusses on measuring radiation dose to the blood pools’ circulating lymphocytes as they pass through the target such as in the aorta and are treated as a normal tissue due to their vulnerability to radiation [8]. The lungs, heart, and blood arteries are examples of organs with large blood volumes which are considered in this model as they reflect immune cells affected by RT [4] (Fig. 1).

Selecting the appropriate radiation modality is essential for reducing the immunosuppressive effect of RT, for patients with thoracic cancer where administration of immunotherapy shows promising treatment outcomes in selected patients [9]. Due to its accessibility and proven effectiveness, photon therapy is widely used in the management of thoracic malignancies, yet is limited in terms of sparing nearby healthy tissues such as lymphoid organs from unnecessary radiation [10]. Proton therapy has the potential to reduce RIL due to its dosimetric characteristics [11]. Optimising treatment plans and improving overall survival for patients with thoracic cancer requires an understanding of the comparative effects of both.

The aim of this scoping review is to determine whether radiation quality (protons or photons) influences RIL in thoracic malignancies, focusing specifically on lung and oesophageal cancers. Lymphomas were omitted from this review due to the known complexity of the biological mechanism of lymphopaenia in lymphomas such as angioimmunoblastic T-cell lymphoma relative to solid tumours [11]. The objectives of this review are to outline the methodologies used to measure lymphopaenia and to investigate whether patients with RIL have a poorer overall survival than those without. Additionally, this review will compare the dose to lymphoid organs such as bone marrow when treating with protons or photons for thoracic malignancies.

## Methods

### Search strategy for identification of studies

**Fig. 2:**
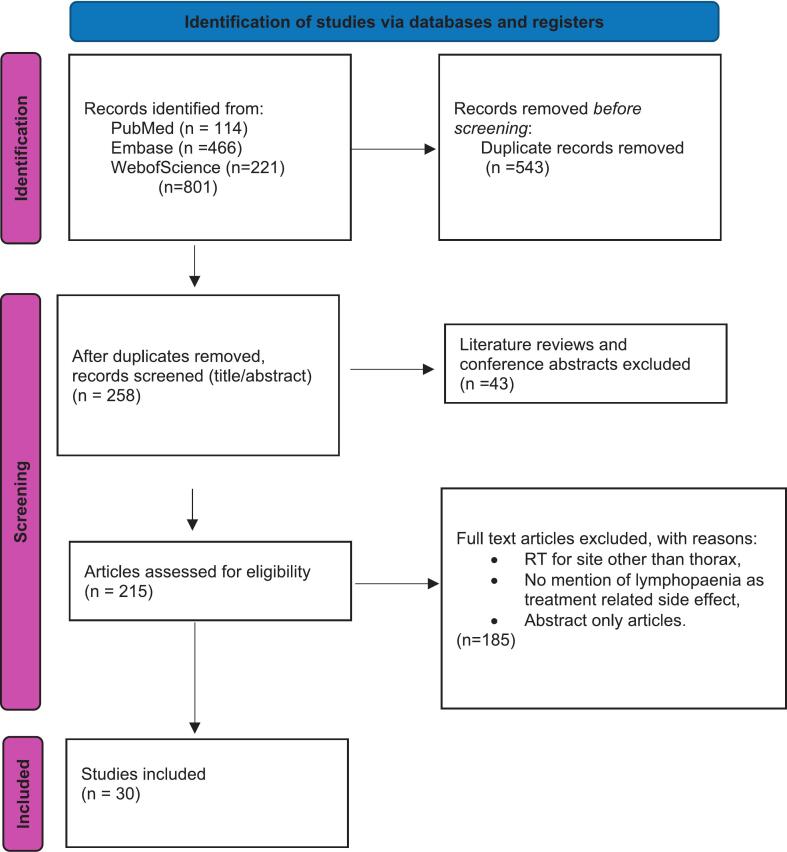
PRISMA diagram.

The search strategy used a combination of terms to identify articles using the Preferred Reporting Items for Systematic Reviews and Meta-Analyses (PRISMA) methodology. Databases searched included PubMed, Embase and Web of Science. The following search terms were used: (‘photon therapy’ OR ‘VMAT’ OR ‘IMRT’) AND ‘Lymphopaenia’ AND (‘oesophagus’ OR ‘oesophageal cancer’ OR ‘esophagus’ OR ‘esophageal cancer’ OR ‘lung cancer’ OR ‘thoracic cancer’ OR ‘NSCLC’). A separate search was carried out for proton therapy including (‘proton therapy) OR ‘passive scattering’ OR ‘active scanning’ OR ‘pencil beam scanning’), along with the other elements of the previous search (Fig. 2).

### Type of studies

This review includes randomised controlled trials, non-randomised controlled trials, (prospective and retrospective), planning studies, comparative studies, non-comparative studies, follow-up studies, and post hoc analyses. Studies in the English language and in full text were included. Included studies were limited to the past ten years to ensure relevance to current practice. Conference abstracts and literature reviews were excluded.

### Type of participants

Patients receiving radiation therapy to the thorax, with and without chemotherapy and/or surgery were included, as were lung and oesophageal cancers of any stage. Participants receiving photon or proton therapy were included, with varying fractionation schedules.

### Type of outcomes

The outcomes detailed in this review are the incidence and severity of RIL, overall survival (OS) for patients with and without RIL, and radiation dose to lymphoid organs, where reported.

## Results

Thirty studies were included in this scoping review (Fig. 2). Twelve studies were focused on lung cancer. Of these 8 were specific to non-small cell lung cancer (NSCLC), 1 to small cell lung cancer (SCLC) and 2 to all lung cancer. Eighteen studies related to oesophageal cancer and one study included patients with any of SCLC, NSCLC or oesophageal cancer.

**Measures of Lymphopaenia**

The most commonly used method to measure the presence of lymphopaenia was the Common Terminology Criteria for Adverse Events (CTCAE), version 4.0 or 5.0, which was utilised in 20 of the 30 papers included [2], [3], [8], [11], [12], [13], [14], [15], [16], [17], [18], [19], [20], [21], [22], [23], [24], [25], [26], [27], [28] (Table 1). Both CTCAE versions 4.0 and 5.0 define lymphopaenia grading based on absolute lymphocyte counts (ALC) as follows: Grade 1 is characterised by ALCs between 0.80–1.00 × 10^9^ cells/L, Grade 2 is defined as ALCs between 0.50–0.80 × 10^9^ cells/L, Grade 3 as ALCs between 0.20–0.50 × 10^9^ cells/L, and Grade 4 by ALCs of less than 0.20 × 10^9^ cells/L [2], [3], [8], [11], [12], [13], [14], [15], [16], [17], [18], [19], [20], [21], [22], [23], [24], [25], [26], [27], [28]. Other papers simply measured the ALC values before, during and following treatment to evaluate the lymphocyte nadir [10], [29], [30], [31], [32], [33].

## Impact of treatment regimens on incidence and severity of lymphopaenia

Only one included study [5] did not report chemotherapy in their treatment regimen. The most commonly reported chemotherapy agents were various combinations of platinum (Cisplatin, Nedaplatin, Carboplatin), taxanes (Docetaxel, Paclitaxel), Fluorouracil, Pemetrexed, and Etoposide.

In 13 included studies, patients were treated with either photon or proton beam therapy [3], [5], [11], [12], [13], [14], [16], [22], [24], [25], [30], [34], [35]. In the remainder, they were treated with photon therapy only (either 3DCRT or IMRT).

The impact of chemotherapy on lymphocyte count is complex [36] and can depend on type and regimen of chemotherapy [18]. For example, it has been noted that some chemotherapy agents can selectively deplete specific lymphocyte subsets, while others can induce immunogenic cell death, which can potentially enhance anti-tumour immunity [29]. Evidence from included studies in this scoping review suggests that indeed the inclusion of concurrent chemotherapy is associated with a sharp decline in lymphocyte count during RT [18], [28]. For example, Grade 4 lymphopaenia was recorded in 38.8% (n = 21) of patients with oesophageal cancer following radiation therapy, in a study by Newman et al. [19], while Ni et al. reported that 13.8% (n = 16) of their included patients with oesophageal cancer experienced Grade 4 RIL [20]. These studies used the CTCAE grading system to assess the severity of lymphopaenia. A progressive decrease of absolute lymphocyte count over the course of six weeks of treatment was further recorded by Xu et al., who reported that the cumulative rates of Grade 3 and Grade 4 lymphopaenia in patients with oesophageal cancer reached 64.7% (n = 282), and 23.6% (n = 103), respectively [27].

**Radiation therapy modality and lymphoid organ doses**

Several factors contributed to lymphopaenia recorded in included studies, including radiation therapy modality. Lymphopaenia recorded in patients receiving photon therapy or proton therapy varied, with one comparative study in lung cancer reporting Grade ≥ 3 lymphopaenia in 47% (n = 33) of patients receiving intensity modulated proton therapy (IMPT) and 67% (n = 134) receiving intensity modulated radiation therapy (IMRT), with lymphopaenia graded according to CTCAE 5.0(13). Participants in this study received concurrent Carboplatin or Cisplatin with or without adjuvant Durvalumab. Another study reported a 45.2% (n = 114) incidence of Grade 4 lymphopaenia in patients receiving IMRT, compared to 22.6% (n = 57) in those receiving PBT, supporting the potential of proton therapy for lymphopaenia reduction [30]. However, the specific chemotherapy regimen used in this study was not reported.

Comparative studies of PBT versus photons in oesophageal cancer indicated a lower incidence of Grade 4 lymphopaenia with PBT compared to photon therapy. Shiraishi et al. [24] reported an incidence of 40.4% (n = 55) with IMRT and 17.6% (n = 24) for PBT, mainly attributed to the lower heart dose with PBT. Patients in this study were treated with neoadjuvant chemoradiation with or without induction chemotherapy. However, this difference in incidence did not lead to an overall survival benefit. Another study described how PBT significantly reduced the occurrence of Grade 4 lymphopaenia compared to photon-based therapy (p < 0.001), but also contributed to superior overall survival [11]. Patients were treated with chemoradiation using 5FU or Capecitabine and a taxane or Carboplatin and a taxane or 5FU and Oxaliplatin. The superior reduction in lymphopaenia was attributed to significantly lower doses to the lungs (V5Gy, V20Gy, V30Gy and mean lung dose) as well as the heart (V5Gy, mean heart dose) and vertebral bodies (V5Gy, V20Gy, mean dose) from PBT compared to IMRT. Another study reported that mean lung dose was reduced by 21.9% and lung volume exposures at 5 Gy, 10 Gy, and 20 Gy were decreased by 47.9%, 36.4%, and 12.1%, respectively, when using PBT compared to VMAT. Additionally, this study also reported a decrease in the mean heart dose by 21.4% and thoracic vertebrae dose by 29.9%, with all results reaching statistical significance (p < 0.05), when comparing proton therapy to VMAT [5].

These study results are explained by the exploitation of the physical properties of proton therapy, including its precise radiation delivery at the Bragg peak and rapid dose fall-off beyond the target [6]. These physical characteristics limit the low-dose bath (V5Gy) to nearby healthy tissues, including the heart and uninvolved lungs for this protective effect [5]. Proton therapy was also reported as decreasing the radiation exposure of the heart’s circulating blood pools, which is closely correlated to the risk of lymphopaenia, reinforcing this benefit of proton therapy [24].

While the mean dose to the heart and lungs was used in a number of studies as a surrogate for radiation exposure to the lymphoid organs, another approach was to calculate the EDIC using a validated mathematical model. So et al. [9] reported that patients with EDIC < 2 Gy had a two year OS of 66.7% (n = 61), those with EDIC between 2 and 4 Gy had OS of 42.7% (n = 39), and those with EDIC > 4 Gy had OS of 16.7% (n = 15), illustrating a substantial variation in patient outcome, based on dose [10].

**Overall Survival**

For the studies focused on lung cancer in this scoping review, the majority of results indicated that overall survival was negatively impacted by the presence of severe lymphopaenia. Abravan et al. [15] reported that > Grade 3 lymphopaenia resulted in worse overall survival than those without. Friedes et al. [2] supported this, reporting that patients in their cohort with Grade 4 lymphopaenia also experienced worse overall survival. They also indicated that patients who had EDIC < 4.7 Gy had improved overall survival. Others have also reported that both EDIC and lymphocyte nadir were significant for overall survival [33]. Yang et al. [8] reported a threshold EDIC of ≥ 2.89 Gy and pre-RT absolute lymphocyte count (ALC) of 2.03 X 10^9^ cells/L. Jing et al. [3] noted that postoperative chemoRT patients with severe lymphopaenia had a median overall survival of 28.4 months compared to 48.3 months in those without, while in a study of 223 patients, 2 year overall survival was reported at 63.4% for those with severe lymphopaenia compared to 79.9% for those with non-severe lymphopaenia [16].

Oesophageal cancer studies yielded similar results, with most indicating reduced overall survival in the presence of severe lymphopaenia. Ni et al. [20] reported 3 year overall survival of 50% for patients who experienced Grade 4 lymphopaenia compared to 66.5% for patients who had Grades 1–3 lymphopaenia, although this was not statistically significant. In comparison, Saito et al. [23] reported 3 year overall survival at 73% for those with Grade 4 lymphopaenia and at 80% for those with Grade 3. Others have reported 3 year overall survival for Grade 4 lymphopaenia as low as 41% and as significantly worse than for patients with Grade 3 [27]. Others have also reported worse overall survival, without specific temporal endpoint reporting [14], [19], [22], [25], [30].

For EDIC analysis, it was reported that an EDIC < 2 Gy resulted in 2 year overall survival of 66.7%, 2–4 Gy an overall survival of 42.7% and 4 Gy of 16.7% [10]. Tian et al. [26] demonstrated that the overall survival of patients with min ALC < 0.41 X10^9^ /L was significantly lower than those with min ALC > 0.43 X 10^9^/L. While not providing a threshold, Wang et al. [29] concurred that a low ALC nadir during radiation therapy was significantly associated with poor overall survival.

Additionally, the time of lymphopaenia occurrence and time to recovery also appear to impact overall survival. Referring to the dynamics of lymphopaenia over time, severe lymphopaenia was reported as typically peaking during RT and gradually improving post-RT. However, delays in lymphopaenia recovery beyond 4–12 weeks after RT were significantly linked to worse overall survival (p = 0.001) [31]. This indicates the possibility of acute and delayed lymphopaenia as predictors of longer term survival in patients with this study having a median follow up of 24 months [31].

## Discussion

This scoping review assessed the effects of radiation modality on the dose to lymphoid organs, overall survival, and the incidence and severity of radiation induced lymphopaenia in lung and oesophageal malignancies. The review emphasises the significance of lymphopaenia, the methods for measuring it, and the dosimetric benefits of proton therapy over photon therapy through synthesising evidence across 30 studies.

RIL is caused by incidental radiation dose to organs which have a very high circulating blood pool such as lung, heart and the major vessels as well as to lymphoid organs such as spleen and bone marrow. Both circulating and resident lymphocyte subpopulations are therefore depleted, leading to reduced infiltration of immune cells into the tumour microenvironment and the tumour itself, impacting on outcome [37]. It should be noted that lymphopaenia effects are not confined to those being treated curatively. Lymphopaenia can also impact on patients with thoracic malignancies being treated with palliative intent, as reported for patients with primary non-small cell lung cancer presenting with brain metastases for which they received stereotactic radiosurgery (SRS). Forty eight percent (n = 60) developed lymphopaenia during treatment which was especially significant as many were receiving immunotherapy within 3 months of SRS and those with lymphopaenia had significantly shorter PFS (2.9 vs 8.5 months) and OS (3.0 vs 19.4 months) than those without [38].

The management of lung and oesophageal cancers with proton beam therapy is complex owing to range uncertainties caused by motion and density changes [39]. However, when compared to photon therapy in this review, proton therapy demonstrated the potential to reduce RIL. The dosimetric characteristics of proton therapy, including its Bragg peak and rapid dose fall-off, reduced the dose to lymphoid tissues, including the heart and bone marrow [5], [6]. For example, one study reported that 45.2% (n = 114) of patients who received photon therapy had Grade 4 RIL, compared to only 22.6% (n = 57) [30]. These results suggest that proton therapy maintains lymphocyte counts during treatment, which may improve the integration of immunotherapy through preserving immune function.

This scoping review includes data that links significant (Grade 4) lymphopaenia with poorer overall survival. There was also a notable difference in overall survival outcomes based on EDIC in some studies. Of note is the 2-year OS for patients with an EDIC < 2 Gy at 66.7% (n = 61), compared to EDIC values between 2 and 4 Gy being 42.7% (n = 39) and with EDIC > 4 Gy at just 16.7% (n = 15) [10]. These findings highlight EDIC’s potential in outcome prediction, albeit a surrogate metric that is determined by radiation dose to organs such as the heart and lungs rather than by measuring the number of circulating lymphocytes. It is acknowledged that this approach also has its limitations as percentages of blood volume allocated to the heart, lungs, great vessels, and small vessels/ capillaries might not accurately represent the body’s blood distribution, and the dose contributions were estimated using average parameters rather than being patient-specific [40]. Moreover, the primary aim of prediction with EDIC is not the prevention of lymphopaenia but an effort to reduce side effects such as cardiovascular effects or pneumonitis, which are strongly linked to radiation doses to the heart and lungs [41]. Prioritising immune preservation in treatment planning is made more difficult due to the absence of lymphopaenia-specific dose volume constraints (DVCs). Given the variation of survival outcomes linked to EDIC, reducing radiation exposure to immune- critical regions may enhance patient outcomes. The use of surrogates, however, highlights a gap in clinical practice, as the DVCs explicitly targeting lymphoid tissues such as the thymus, spleen, and bone marrow are currently absent. Dose to the vertebral body, sternum and ribs were included as the thoracic bones and thoracic bone V5 Gy and V10-50 Gy were significantly associated with lymphopaenia, irrespective of proton or photon delivery [25].

Lymphopaenia also influences other aspects of patients’ outcomes as they progress through treatment. It has implications for patients receiving immunotherapy as it disrupts the immune system’s capacity to achieve an effective anti-tumour response and causes potential resistance to immunotherapy [42]. Lymphocytes, particularly T cells, are important for the effectiveness of immune checkpoint inhibitors which rely on a functioning immune system to promote tumour destruction [43]. Furthermore, a reduction in lymphocyte count, along with other factors such as advanced age, may enhance immune suppression within the tumour microenvironment, which can cause tumour progression, or may represent worse physical health [44]. Although radiation therapy can cause severe and prolonged lymphopaenia, through reducing cytotoxic lymphocytes that reach the microenvironment, combined immuno-radiation therapy can be a promising treatment modality [45]. Radiation-induced tumour damage improves dendritic cell ability to detect tumour cells and triggers T-cell cytotoxic response, thus increasing the effectiveness of immunotherapy [45]. These contrasting concepts must be taken into consideration when planning the treatment of patients using immuno-radiation therapy. Here is where the use of proton therapy could be considered with its potential to spare lymphoid organs and reduce radiation exposure to circulating lymphocytes, preserving immune competency [6].

A limitation of this scoping review is the inability to differentiate between RIL caused by definitive radiation and that caused by chemo-radiation therapy. This is due to the retrospective nature of the majority of studies in this scoping review (n = 28) and a paucity of prospective evidence.

Future prospective research could focus on incorporating immunotherapy considerations into radiation therapy planning and optimising radiation therapy techniques in order to reduce dose to lymphoid tissues, while maintaining tumour control. While proton facilities are increasing in number, proton therapy is still not universally accessible, and the cost effectiveness of its use in minimising RIL would need to be established [46].

## Conclusion

In conclusion, this scoping review highlights the potential of proton therapy in reducing radiation-induced lymphopaenia in lung and oesophageal cancers when compared to photon therapy. Proton therapy may improve treatment outcomes by preserving immune function through lowering radiation exposure to lymphoid tissues. This is particularly important for patients who are receiving immunotherapy as part of their management strategy. Immune preservation and treatment strategies including multimodality treatment approaches can be improved through establishing dose volume constraints to lymphoid organs and incorporating lymphocyte-sparing techniques in radiation therapy planning.

## Declaration of competing interest

The authors declare that they have no known competing financial interests or personal relationships that could have appeared to influence the work reported in this paper.

## Figures and Tables

**Table 1:** Table of included studies.

Article (year)	No. patients	Study type	Treatment site	Treatment	RT	Recorded presence of lymphopaenia	Lymphopaenia measurement	Lymphoid organ dosimetry	Outcome (overall survival)
Cortiula et al. (2024)	271	Retrospective analysis of prospectively collected registry of patients with Stage III NSCLC in 4 centres	NSCLC	Concurrent Carboplatin or Cisplatin −RT +/-adjuvant durvalumab	IMPT, IMRT	Lymphopaenia grade ≥ 3 47% vs 67% in IMRT and IMPT respectively	Lymphopaenia graded according to CTCAE 5.0	Bone marrow- V20Gy	Lymphopaenia not associated with worse OS (p = 0.11)
Davuluri et al. (2017)	504	Retrospective cohort study in a single centre	Oesophagus	Neoadjuvant or definitive chemo-RT (combinations of taxanes, 5FU and platinum)	IMRT, PBT	Incidences of grade 1, 2, 3, and 4 ALC nadir during CRT: 2%, 12%, 59%, and 27%, respectively at 36 *m*	Weekly absolute lymphocyte count (cells × 10^3^/μL) measured during and prior to CRT. Lymphopaenia graded according to CTCAE 4.0	Bone marrow 10 Gy −30% reduction in PBT. Mean body dose	Grade 4 = worse OS. PBT did not improve OS.
Abravan et al. (2020)	901 lung 305 oes.	Retrospective cohort study in a single centre	NSCLC, SCLC, Oesophagus	Definitive RT/ sequential or concurrent chemo-RT.	Photon therapy-not specified	Lung: 82% Grade 2 < during RT; 44% Grade 3, 10% Grade 4. Oesophageal: 91% Grade 2<, 56% Grade 3, 16% Grade 4 during rt.	Full blood counts available pre-RT and during Rt (lymphocyte count × 10^9^/L) Lymphopaenia graded using CTCAE 4.0	Mean dose to heart, lung and thoracic vertebrae −V20Gy	Oesophageal Grade 4 lymphopaenia –worse OS (p < 0.001) Lung Grade > 3 lymphopaenia worse OS (p = 0.002)
Chen et al. (2024)	734	Retrospective cohort study in a single centre	Oesophagus	Concurrent chemo-RT	PSPT and IMRT	Incidence Grade 4 in IMRT arm: 45.2% vs PBT arm: 22.6%,	Baseline and at least 3 weekly absolute lymphocyte count × 10^3^/ μL	Discusses reducing dose to bone marrow, but no constraints stated	OS varied significantly by Grade 4 RIL incidence (P = 0.0018)
Ebrahimiet al. (2021)	10	Prediction modelling study	Oesophagus	Chemo-RT	IMRT, PSPT, IMPT	3 patients had grade 2 lymphopaenia, 4 had grade 3, and 3 had grade 4	Models fitted with weekly ALC. (cells × 1000/μL). Grade 4 lymphopaenia and grade 3, grade using CTCAE 5.0, are defined as ALC, 200 cells/µL and ALC, 500 cells/ µL, respectively.	Average mean body dose- 7.46, 4.84, and 3.85 Gy for IMRT, PSPT, IMPT respectively. Model I: linear relationship between lymphocyte survival and voxel-by-voxel dose. Model II: assume lymphocytes deplete exponentially with total dose.	Not stated
Friedes et al. (2024)	228	Retrospective cohort study in a single centre	NSCLC	Chemo-RT +/- consolidative immunotherapy Carboplatin/Paclitaxel or Cisplatin/Etoposide or Docetaxel and Pemetrexed containing regimens	Not stated	Grade 1: 26 patients, G2: 143, G3: 424, G4: 170	Baseline lymphocyte count and throughout RT (minimum recorded value during RT = ALC nadir) Lymphocyte count × 10^9^ cells/L) Lymphopaenia was graded per CTCAE v5.0	Immune cells – median dose 4.7 Gy	Patients with effective dose to immune cells < 4.7 Gy had longer OS (p = 0.002) G4 lymphopaenia: worse OS (p < 0.001)
Jing et al. (2021)	170	Retrospective cohort study in a single centre	NSCLC	Postoperative RT Platinum-based chemotherapy delivered peri RT	3D-CRT, IMRT, VMAT, IMPT, PSPT	Grade 3 to 4 lymphopaenia: 54.8%	ALC collected pre-RT, during RT and 1-month post RT. (ALC × 10^3^/ µL). Lymphopaenia graded using CTCAE 5.0. SRIL: lower tertile value as ALC < 0.37 x 10^3^/ul	Total mean lung dose was significantly correlated with severe RIL (no threshold dose reported)	Patients with severe RIL median OS: 28.4 m compared to 48.3 m in non-severe RIL patients
Kang et al. (2022)	272	Retrospective cohort study in a single centre	NSCLC	Chemo-RT Docetaxel/Cisplatin or Paclitaxel/ Carboplatin	3D-CRT, VMAT	Severe lymphopaenia: 84%, 48%, and 10%, during RT, from 0-4 weeks and 4–12 weeks post CRT, respectively.	ALC at baseline, throughout and after RT were recorded from differential blood count data. Severe lymphopaenia: (ALC nadir < 500 cells/mm3)	Thoracic vertebrae, heart and lung dose not stated	Delayed lymphopaenia: inferior OS (p = 0.001)
Kim et al. (2021)	223	Retrospective cohort study in a single centre	Lung	Definitive concurrent chemo-RT Paclitaxel/Cisplatin or Paclitaxel/Carboplatin or Etoposide/Cisplatin	PBSPT, IMRT	Increased quartiles of PTV < 370, 370–530, 530–790, and > 790 cm3 increased risk SRL- IMRT vs. PBSPT: 18.0%, 28.3%, 43.1%, and 53.2% vs. 20.0%, 14.3%, 14.3%, and 0.0%, respectively	Peripheral blood counts taken every week during CCRT according to CTCAE version 5.00.	Lung V5Gy. Bone marrow, thymus, spleen, and lymph nodes doses not stated.	2-year OS rates: 63.4% and 79.9% for severe RILand non-severe RIL respectively (p = 0.002)
Li et al. (2019)	220	Retrospective analysis of prospectively collected registry in a single centre	Oesophagus	Neoadjuvant CRT	3DCRT, IMRT	Incidence grade 0,1,2,3 and 4 lymphopaenia during CRT: 1.8%, 6.8%, 31.4%, 38.2% and 21.8%, respectively. median ALC declined to 1.0, 0.70, 0.55, 0.46, and 0.50 × 109/L from weeks 1 to 5	ALC determined before, during and 1 month post CRT. Lymphopaenia measured using CTCAE version 4.00	Not in study design	3-year OS rate: 70.1%. Grade 4 lymphopaenia: significantly worse OS (p < 0.05)
Li et al. (2024)	215	Retrospective cohort study in a single centre	NSCLC	CCRT, SCRT Docetaxel/Cisplatin/ Carboplatin or Gemcitabine/ Cisplatin/ Carboplatin or Pemetrexemed/ Cisplatin/ or Carboplatin Paclitaxel/Cisplatin/ or Carboplatin alone	IMRT	Not stated	Lymphocyte reduction graded weekly with CTCAE 5.0. SRL: (ALC) of ≤ 0.2 × 103 cells/μL.	Not in study design	OS better in patients without SRL (in the CCRT group, P = 0.01 and in the SCRT group, P = 0.08)
Newmanet al. (2020)	54	Retrospective cohort study in a single centre	Oesophagus	Chemo-RT Carboplatin/ Paclitaxel or Other platinum-based compounds	IMRT, 3DCRT	21 Grade 4 (38.8%) of lymphopaenia	Used CTCAE 4.0. defines grade 4 lymphopaenia: ALC < 200 cells/µL.	Splenic dosimetric parameters (in ccs) from volumes 5–30 Gy. Absolute vertebral volumes (ccs) receiving 10–40 Gy. Peripheral lymphocytes, bone marrow, circulating lymphocytes doses not stated	RIL associated with decreased OS.
Ni et al. (2022)	116	Post hoc analysis of randomized controlled trial	Oesophagus	Post-op RT or chemo-RT Paclitaxel/Cisplatin or Paclitaxel/Nedaplatin	Photon therapy- not specified	During RT 13.8% had G4 lymphopaenia	Used CTCAE 4.03.	Thoracic marrow V20 ≥ 75%; thoracic vertebral body V20 ≥ 71%. Heart (V15 ≥ 40%)	3-year OS between grade 4 RIL and grade 1–3 (50.0% vs 66.5%, p = 0.095)
Nowicka et al. (2024)	306	Retrospective cohort study in three centres	NSCLC	RT +/- concurrent chemo	IMRT, VMAT	ALC loss range: 11 to 78%. Median % ALC during first 45 days: 52.2% and higher in CRT than RT.	At least 3 blood morphology test- 1 at baseline.	Heart, sum of lungs and body: IMRT- higher lung exposure (15 Gy < ) and bigger heart volume low and high dose (1–55 Gy)	Not stated
Sang –Oh et al. (2023)	132	Retrospective cohort study in a single centre	Oesophagus	RT or Chemo RT Paclitaxel/Carboplatin or Capecitabine/5FU/ Cisplatin	PBT	Grade 3: 22%, no grade 4	Not stated	Cardiopulmonary dose sparing.	No cases of grade 4 or above lymphopaenia observed, Grade 3 identified in 22% of included patients
Patel et al. (2024)	12	Retrospective cohort study in a single centre	NSCLC	Chemo-RT	IMRT, IMPT	Not stated	Not stated	Calculate EDIC using mean lung dose, mean heart dose, body volume, using Jin et al (PMID: 34944813) and Ladbury et al’s (PMID: 31175902) models	Modelled OS2 rate was found to be significantly lower with IMRT than IMPT using EDIC (median 63% vs 71%; P = 0.03)
Qiu et al. (2023)	381	Retrospective cohort study in a single centre	Oesophagus	RT or ChemoRT Paclitaxel/Platinum or 5FU/Platinum or Docetaxel/Platinum	IMRT, 3DCRT	83% grade 3 or more lymphopaenia	Graded using CTCAE 4.0 EDIC calculated: estimates the dose to immune cells by using the radiation dose to circulation blood as a surrogate		low-EDIC significantly improved the OS of patients when compared with high-EDIC (P = 0.003)
Routmanet al. (2019)	144	Retrospective cohort study in a single centre	Oesophagus	RT or ChemoRT Carboplatin/Paclitaxel	3DCRT, IMRT, PBSPT	Proton: grade 1/2––62.5%, grade 3/4:-37.5% Photon: grade 1/2––44.1%, grade 3/4––55.9% Rate G4L not different for 3DCRT versus IMRT	Graded using CTCAE 4.0 with G4 defined as ALC < 200/mm3	Bone marrow, circulating lymphocytes –dose reduced in PRT. Lung receiving ≥ 5 Gy: risk of lymphopaenia	Grade 4 lymphopaenia: decreased OS
Saito, et al. (2018)	61	Retrospective cohort study in a single centre	Oesophagus	Chemo-RT Cisplatin/5FU or Docetaxel/Cisplatin/5FU	Not stated	79%: Grade 4 lymphopaenia, other 21%: Grade 3	ALC at nadir: lowest value recorded during CRT. Graded using CTCAE 4.0 with grade 4 (ALC < 0.200 × 109/I)	Increase of 1 Gy in mean splenic dose: 2.9% decrease in nadir ALC. mean splenic dose was 2.87 (0–31.77) Gy.	No statistically significant difference in OS for grade 3 or lower lymphopaenia (3 year OS = 80%) and those with G4L (3 year OS = 73%) (p = 0.68).
Shiraishi, et al. (2018)	480	Retrospective cohort study in a single centre	Oesophagus	Neoadjuvant chemo-RT+/- induction chemo	IMRT, PBT	Grade 4 lymphopaenia during CRT: 40.4% for IMRT, 17.6% for PBT. (p < 0.0001)	Graded using CTCAE 4.0	Circulating blood dose: large pools of blood in heart: enhanced risk of lymphopaenia. Dosimetric differences of heart in PBT to IMRT leads to less lymphopaenia.	grade 4 lymphopaenia only trended towards significance for poorer OS (P = 0.09)
So et al. (2020)	92	Retrospective cohort study in a single centre	Oesophagus	Neoadjuvant chemo-RT Paclitaxel/Carboplatin	3DCRT, IMRT	EDIC was significantly correlated with lymphocyte nadir (p < 0.01)	Blood tests including Lymphocyte count during neo chemo-RT were retrieved. Lymphocyte nadir: lowest ALC among set of lymphocyte values during chemo-RT and at 2 months post chemo-RT	EDIC calculated as a function of integral doses to lung, heart, and total body with verified mathematical model. mean EDIC was 2.8 Gy (range, 0.6–4.4	lymphocyte nadir was a significant independent factor for shorter OS (hazard ratio = 0.63; P < 0.001) The 2–year OS rates were 66.7%, 42.7%, and 16.7% for EDIC < 2, 2 to 4, and > 4 Gy, respectively
Sumiya et al. (2021)	69	Retrospective cohort study in a single centre	Oesophagus	Chemo-RT 5FU/Cisplatin	3DCRT, PSBT	Not stated	Toxicities evaluated based of CTCAE 4.0	Lung V5 and heart V50 and RT to thoracic bone-likely significantly lower during PBT than 3DCRT. Vertebral body, sternum and ribs included as thoracic bones: V5 and V10–50: associated with lymphopaenia. vertebral volume/mean body dose ≥10 Gy correlated with G4 RIL.	Better OS for grade ≤ 2 compared with grade 4 lymphopaenia. OS was better in the patients with a high ALC than in those with a low ALC (2-year OS rate: 79.9% vs 64.0%), difference not significant (p = 0.083)
Tian et al. (2023)	110	Retrospective cohort study in a single centre	Oesophagus	RT or ChemoRT Cisplatin or Nedaplatin +/- Docetaxel or Capecitabine Docetaxel+/- Cisplatin	IMRT	Incidence of lymphocyte nadir G1, G2, G3 and G4 MinALC during RT: 0.91%, 9.09%, 62.73% and 27.27%, respectively.	Min value of ALC during RT: lowest point of lymphocytes (MinALC). Graded using CTCAE 5.0. Grade 1: 0.8 × 10^9/^L, grade 2: 0.50.8 × 10^9^/L, grade 3: 0.2–0.5 × 10^9^/L, grade 4: 0–0.2 × 10^9/^L.	Bone marrow, thymus, and spleen. Dose not stated.	KM survival analysis showed: OS of patients with MinALC ≤ 0.41 × 10^9/^L, was significantly lower than that of patients with MinALC > 0.43 × 10^9^/L. Patients with G4 lymphopaenia during CRT: worse OS than those without G4: median 34.7 and 63.1 months, respectively
Wang et al. (2024)	105	Secondary analysis of data from a single-institution, open-label, non-blinded, phase 2 randomized trial	Oesophagus	Chemo-RT 5FU or Capecitabine/Taxane or Carboplatin/Taxane or 5FU/Oxaliplatin	IMRT, PBT	44 patients (42%) experienced G4 RILat a median of 28 days after starting concurrent CRT. limiting dose scatter by PBT significantly reduced the incidence of G4 RIL	Grade 4 lymphopaenia graded using CTCAE 5.0	Cardio-pulmonary system: Lung V5Gy, V20Gy, V30Gy, mean dose: (P = 0.001, P = 0.001, P = 0.017, P < 0.001, respectively), heart V5Gy, mean dose (P = 0.008, P = 0.002 respectively), vertebral body V5Gy, V20Gy, mean dose: (p = 0.002, p = 0.007, p < 0.001, respectively)	OS rate was 86.4% at 12 months.
Wang et al. (2020)	189	Retrospective cohort study in a single centre	Oesophagus	Definitive RT or Definitive ChemoRT Platinum/5FU or Platinum/Taxane	IMRT, 3DCRT	Not stated	ALC values were assessed before, weekly during RT, and 1 month after the end of RT.	High heart V10 (P = 0.003), and high heart V20 (P = 0.028) were associated with low ALC nadir during RT	A low ALC nadir during RT was significantly associated with poor OS.
Wong et al. (2021) −	10	Retrospective treatment planning study	Lung	RT	VMAT, PBS proton single-field plans	Not stated	Not stated	Proton: reduced mean lung dose (by 21.9%), lung V5, V10, V20 (by 47.9%, 36.4%, 12.1%, respectively), mean heart dose (by 21.4%) and thoracic vertebra dose (by 29.2%) (P < 0.05)	Potential for PBT to improve OS by sparing dose to the heart, limiting dose to additional lymphopaenia-related organs, such as the lungs and thoracic vertebra.
Xu et al. (2021) −	436	Retrospective analysis of prospectively collected registry in a single centre	Oesophagus	Chemo-RT Cisplatin/Taxane or Cisplatin/5FU or Taxane or Platinum-based compound	3DCRT, IMRT	Median ALC declined from baseline value:1.80 × 10^9^/L to 0.80, 0.50, 0.40, 0.30, 0.30, and 0.35 × 10^9^/L from weeks 1 to 6 during CRT, cumulative incidence of G0, G1, G2, G3, and G4 RIL during CRT were 0.2%, 1.6%, 9.9%, 64.7%, and 23.6%, respectively	ALC values collected prior to and during CRT and 1 month after. Treatment-related lymphopaenia was graded using CTCAE version 4.0.	G4 RIL (n = 103) had significantly higher V5,V10,and V20 of the lung, higher V10,V20,and V30 of the heart and higher thoracic vertebrae V10 and V20	3-year OS rates: 41%. G4 lymphopaenia was significantly associated with worse OS (p < 0.001) than the G0–3 lymphopaenia group.
Yang et al. (2023)	517	Retrospective cohort study in a single centre	NSCLC	Chemo-RT +/- immunotherapy Paclitaxel/ Carboplatin or Durvalumab or Pembrolizumab or Nivolumab	3DCRT, IMRT	EDIC ≥ 2.89 Gy and pre-RT ALC < 2.03 × 10^9^ cells/L- higher incidence of sever RIL. incidences of severe lymphopaenia in the high-intermediateand low-risk groups were 90.1%, 77.1%, and 52.3%, respectively (P < 0.001)	Lymphocyte counts of 0.80–1.00 × 10^9^ cells/L, <0.80–0.50 × 10^9^ cells/L, <0.50–0.20 × 10^9^ cells/L, and < <0.20 × 10^9^ cells/L were defined as grade G1, G2, G3, and G4 lymphopaenia according to CTCAE 4.03. severe lymphopaenia: G3<	Effective dose to immune cells (EDIC): calculated as a function of the mean heart dose (MHD), mean lung dose (MLD), mean body dose (MBD), and number of fractions.	Worse OS for high risk group (EDIC ≥ 2.89 Gy and pre-RT ALC < 2.03 × 10^9^ cells/L)
Yu et al. (2021)	503	Retrospective cohort study in a single centre	SCLC	Chemo-RT (Sequential or concurrent) Platinum/Etoposide	3DCRT, IMRT	Rates of grades 0, 1, 2, 3, and 4 lymphopaenia were 1%, 9%, 30%, 55%, and 5%, respectively. EDIC was correlated with lymphocyte nadir under univariate (p < 0.001) and multivariable linear regression (p < 0.001)	Lymphocyte nadir was defined as the minimum lymphocyte count during thoracic radiation therapy, collected retrospectively.	EDIC is an estimated dose to immune cell by using the dose to circulation blood pool as a surrogate (approximated from mean lung, heart dose and body dose), median EDIC was 7.34 Gy (range 1.81–18.76 Gy)	Multivariable analysis showed EDIC (p = 0.005) and lymphocyte nadir (p = 0.003) were both significant for OS.
Zhou et al. (2019)	289	Retrospective cohort study in a single centre	Oesophagus	Concurrent Chemo-RT +/- consolidation Chemo Cisplatin/Docetaxel	3DCRT, IMRT	During CCRT, a total of 89 patients (31%) had treatment-related lymphopaenia.	ALC < 200 cells per mm^3^ (Grade 4, CTCAE 4.0) during CCRT weeks 1–6 was defined as treatment-related lymphopaenia.	Circulating lymphocytes received RT dose while passing through the lung- higher mean lung dose = lymphocyte nadir. Unintentional RT to lymphoid sites: bone marrow, thymus, spleen, lymph nodes may contribute to RIL.	Cancer-related death were more frequently observed in treatment-related lymphopaenia group than those without (58.4% vs. 39.6%). Survival analysis not performed due to short follow-up time.

## Appendices

Abbreviations: RIL= radiation-induced lymphopaenia

CRT=ChemoRT, ALC= absolute lymphocyte count

EDIC= Effective dose to immune cells
